# Older Amyloid Beta as a Candidate Blood Biomarker of Early Cognitive Decline in the Elderly—A Preliminary Study

**DOI:** 10.3390/cimb47030203

**Published:** 2025-03-18

**Authors:** Oliwia McFarlane, Mariusz Kozakiewicz, Kornelia Kędziora-Kornatowska, Anita Gałęska-Śliwka, Milena Wojciechowska

**Affiliations:** 1 Nicolaus Copernicus University in Toruń, Ludwik Rydygier Collegium Medicum in Bydgoszcz, Faculty of Health Sciences, Department of Law and Health Policy, Świętojańska 20, 85-077 Bydgoszcz, Poland; 2 Nicolaus Copernicus University in Toruń, Ludwik Rydygier Collegium Medicum in Bydgoszcz, Faculty of Health Sciences, Department of Geriatrics, Curie-Skłodowskiej 9, 85-094 Bydgoszcz, Poland

**Keywords:** Alzheimer’s disease, cognition, dementia, MCI, neurodegeneration, plasma, amyloid beta plaques and fibrils

## Abstract

(1) Background/Objectives: The pathogenic process of Alzheimer’s disease (AD) is known to begin decades before its clinical onset. This period, although imperceptible to the patient, encompasses a gradual neuronal loss. The first symptoms of dementia, often classified as mild cognitive impairment (MCI), in many cases converts into incipient AD, but can also remain stable or even reverse to cognitive norm. An easy and fast blood-based method of identifying patients at risk of conversion to AD would allow for the application of disease-altering therapies. This preliminary study focuses on the identification and assessment of the relationship between plasma amyloid beta (Aβ) and cognitive performance in older Polish adults with respect to its adequacy as a biomarker of an early cognitive deterioration. (2) Methods: The preliminary research sample consisted of 230 participants, 109 females and 121 males, aged 65 plus. The association between plasma Aβ concentrations with cognitive status, gender, and age were assessed. The analyses were conducted in three categories of cognitive performance: cognitive norm, mild cognitive impairment, and mild dementia, based on results of the Mini-Mental State Examination (MMSE) and functional tests. (3) Results: No significant differences in plasma Aβ levels for different cognitive statuses were identified. No significant differences were found in Aβ levels based on age or gender. (4) Conclusions: In order to thoroughly explore the power of research on plasma Aβ with respect to early cognitive deterioration, further prospective studies are required.

## 1. Introduction

Neurodegenerative disorders, such as Alzheimer’s disease and other dementias, constitute the main reason for disability and dependency in the elderly, significantly lowering the quality of life of older people. Due to rapid population aging, these disorders impose a growing burden upon patients, their families, and communities. Estimation of the worldwide prevalence of dementia in 2019 was 57 million people, with prognosed increase to 153 million by 2050 [1].

Macroeconomic implications of Alzheimer’s disease and other dementias are severe. They are projected to cost the world economy 14,513 billion international dollars, equivalent to 0·421% of annual global GDP [2].

Subsequent to the discovery of the first detection techniques of dementia biomarkers, there has been an increased scientific interest, driving research focus in this area. Today, the basic established Alzheimer’s disease (AD) biomarkers, Aβ-binding ligands used in positron emission tomography (PET), Aβ42, total-tau (ttau), and phosphor-tau (ptau) in cerebrospinal fluid (CSF) [3], reflect several key pathophysiological characteristics of the condition, and convey diagnostically appropriate data, including concerning its preclinical phase. In spite of the undeniable usefulness of these markers, the fact that they are either invasive or time-consuming and expensive to identify poses substantial limitations to their application [4]. Therefore, the last 20 years has witnessed a significant number of studies towards the discovery of biomarkers in peripheral blood. Their identification is associated with increased time- and cost-efficiency, reduced invasiveness, and increased patient acceptance, leading to ease of implementation with large populations [5,6], and they meet many of the requirements set for an ‘ideal’ biomarker. Also, with multiple potential contexts for their application, including clinical trials, primary care screening, diagnostics, predictive risk, disease monitoring, and therapy response tracking [7], they could revolutionize dementia diagnosis and treatment [8]. Even though AD currently remains incurable, there is little doubt that a minimally invasive blood-based biomarker for screening in the preclinical phase would represent an essential element of future therapeutic approaches. At the minimum, it could commence a tiered diagnostic course [6,9,10]. Consequently, there has been a vast scientific interest in the development and validation of peripheral blood biomarkers [5,9,11,12]. However, despite the enormous expansion of this area, the field has been impaired by both reproducibility and reliability issues, as well as lack of a clear path for shifting basic discovery to clinical application. Nevertheless, several candidate biomarkers have demonstrated potential for clinical utilization in AD, and/or promise for future implementation [8]. Amyloid-β is amongst those biomarker candidates.

Aβ is formed by proteolytic split of amyloid precursor protein (APP), generated through sequential processing by β- and γ-secretases, and secreted to CSF. Amyloid beta encompasses proteins composed of 36–43 amino-acids, being a main constituent of amyloid plagues, and an original AD pathogenesis factor according to the amyloid cascade hypothesis. The basic function of Aβ is not known [13]. There is data suggesting that a lack of this protein does not lead to loss of any biological functions, although some potential tasks of Aβ assume kinase enzyme activation [14], antioxidative function [15], or cholesterol transport regulation [16]. Latest findings indicate that Aβ can play a role in fighting certain diseases, attacking pathogenes in mice and worms [17]. Two primary pathological isoforms of Aβ exist: Aβ40 and Aβ42, identified by their amino acid length. The most toxic form, dominant in brain accretion, is the 42- acid isoform. It is characterized by an amplified ability to form oligomers and fibrils—the major component of amyloid plaques—than its shorter counterpart [18]. Intracellular concentration of certain divalent cations can also promote fibril formation [19] and subsequent onset of Alzheimer’s malignancies.

Since Aβ penetrates the blood–brain barrier, any levels detected in circulation derive both from the brain and the periphery. As regards Aβ and its tissue reactions, blood detection means have largely involved the use of the enzyme-linked immunosorbent assay (ELISA), which was also utilized in this study. Improvements in the sensitivity of the ELISA for both Aβ40 and Aβ42 have allowed for the measurement and quantitative analysis of Aβ in human blood [20]. Aβ42 seems to be a logical candidate for a plasma biomarker that could enable identification of patients susceptible to developing AD. However, until now, research has brought conflicting results regarding its usability. In general, studies on plasma Aβ42 as a biomarker reflecting brain amyloid pathology have demonstrated some disappointment due to no or scarce alterations and broad overlaps in both Aβ42 and Aβ40 concentrations between patients and controls [21]. Recent research suggests that conflicting results may also be an effect of the Aβ expression by cells in peripheral tissues, such as platelets [22], skin fibroblasts [23], or skeletal muscles [24], which boost the total Aβ blood concentration. Therefore, available findings have not yet allowed the use of this method for a differentiating diagnosis [25], and have highlighted the need for further research efforts in this field. It has lately been suggested that a hypersensitive single molecule array (SIMOA) might enable more accurate detection of Aβ in plasma. Also, the development of fluorescent tools (such as a multichannel fluorescent sensor array) as alternative sensing techniques [26] may contribute to future advances in this area.

## 2. Material and Methods

The preliminary study was conducted on inpatients of the Clinic of Geriatrics of Collegium Medicum in Bydgoszcz, Nicolaus Copernicus University in Toruń, Poland. The study followed the adequate institutional and governmental regulations regarding the ethical use of human volunteers and the tenets of the 1975 Helsinki Declaration. The institutional review board approved the study protocol (no KB 505/2014) and all the recruited patients expressed their written informed consent.

### 2.1. Material

The sample was composed of 230 Non-Hispanic White inpatients—109 females and 121 males aged 65 and over at sampling (Table 1). The exclusion criteria are listed below: (1) use of statins, (2) Geriatric Depression Scale result > 5 pt., (3) moderate to severe dementia, (4) brain stroke, (5) acute health condition, (6) other pathological states that might substantially alter cognitive status. Tests were performed in three categories of cognitive functioning: cognitive norm (regarded as controls), mild cognitive impairment, and mild dementia, based on the results of the Mini-Mental State Examination (MMSE) and functional tests (ADL, IADL). The cognitive norm (control) group was composed of 71 patients (33 females and 38 males; average age 77.8); the MCI group was composed of 85 subjects (43 females and 42 males; average age 78.8); the mild dementia category was composed of 74 participants (33 females and 41 males; average age 80.7). The groups were age and gender matched.

### 2.2. Biochemical Analysis

Blood samples were collected by vacuum venipuncture. Heparin plasma was stored at −80 °C in the Department of Geriatrics Collegium Medicum in Bydgoszcz, Poland. Aβ plasma levels were assayed with ELISA immunoenzyme assay (sensitivity: <2.63 pg/mL). Prior to assaying, the samples were kept in room temperature (18–25 degrees Celsius).

### 2.3. Socioclinical Analysis

The evaluation consisted of the chosen parameters (age, gender, socioeconomic status, health status, lifestyle factors) and test results: Geriatric Depression Scale (GDS) [27], Activities of Daily Living (ADL) [28], and Instrumental Activities of Daily Living (IADL) [29]. Cognitive status was determined using the MMSE [30]. The total score of MMSE is 30 points; a score of 30–28 points was regarded as cognitive norm, 27–24 points as MCI, and 23–20 points as mild dementia. The test was administered by trained nurses.

### 2.4. Statistical Analysis

Statistica 10.0. (StatSoft Inc., Tulsa, OK, USA, 2016), R 3.3.0 statistical packet (R Core Team, 2016), and RStudio 1.0 environment (RStudio Team, 2016) were utilized. Not-normally distributed data are shown as median with first and third quartile, and was analyzed with the Mann–Whitney U test or the Kruskal–Wallis test, as applicable. *p*-value < 0.05 was considered to mark statistical significance.

## 3. Results

### 3.1. Socioclinical Analysis

The sample was comparable with regards to social, economic, lifestyle, and health characteristics that might substantially alter cognitive status and increase the risk of developing dementia (i.e., poor education, smoking, obesity, depression, physical inactivity, excessive alcohol intake, and social isolation) [31,32]. In general, the sample was poorly educated (78% of patients had primary to vocational education level), had physical/agricultural employment history (97% with employment history, mainly [71%] as laborers or farmers), and were in declaratively good financial situations (76% described as such). Most (95%) had children. Seventy-six percent declared performing physical activity within the last year, whereas recent involvement in exercise or rehabilitation was reported by 13%. Fourteen percent declared sport activities in the past. The vast majority (89%) reported the ability to climb one floor, while a minority (13%) believed they were able to swim 10 m. Of the patients, 18% had a legal disability title; 87% had normal thyroid function, with subclinical thyroid disease in 13%. Seventy-three percent reported no vision problems. Of the group, 95% reported no treatment for depression. Alcohol use of a few servings/year was reported by 84%. Of the respondents, 97% were independent in basic ADLs, which is consistent with early cognitive decline status.

### 3.2. Aβ42 Plasma Levels

**Figure 1:**
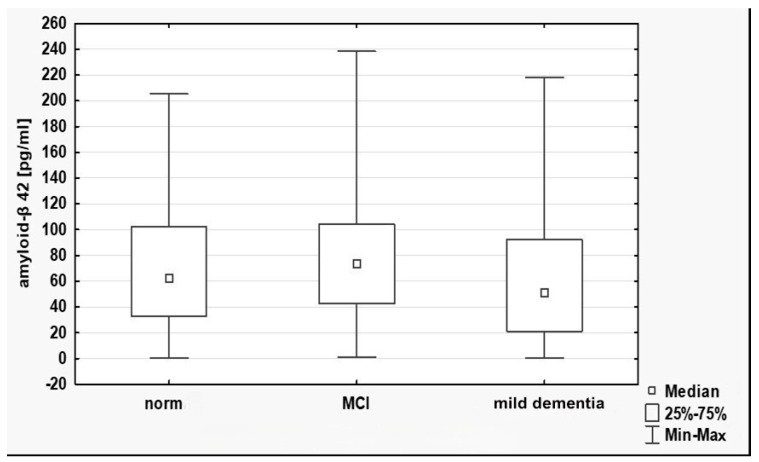
Plasma Aβ42 and cognitive status.

No statistically significant differences in Aβ42 plasma concentrations between the groups were shown (Table 2, Figure 1). No statistically significant differences were identified in Aβ42 concentrations with respect to gender (Table 3). Similarly, no statistically significant differences were identified in Aβ42 concentrations with respect to age.

**Table 2:** Plasma Aβ42 and cognitive status (median [1 quartile–3 quartile]).

	Cognitive Status			ANOVA K-W *p* Level
	Norm	MCI	Mild Dementia	
Amyloid-β 42	62.8 [32.8–102.4]	73.5 [42.4–104.5]	51.6 [21.2–92.6]	0.198

**Table 3:** Plasma Aβ42 and gender (median [1 quartile–3 quartile]).

	Gender		U M-W *p* Level
	Female	Male	
Amyloid-β 42	62.1 [29.0–93.7]	62.8 [31.8–104.0]	0.862

## 4. Discussion

This preliminary study focuses on identification and assessment of the relationship between plasma Aβ42 and cognitive status in older Polish adults with respect to its adequacy as a biomarker of an early cognitive deterioration.

No significant differences in plasma Aβ42 levels between the cognitive status categories were identified. A few hypotheses can account for the high range of observed data variability. A study on factors associated with fluid biomarker levels in a large clinical cohort with subjective cognitive complaints or MCI found certain variables to account for this effect, i.e., inflammatory markers, behavioral risk factors, treatments, comorbidities, and sample handling. However, a large proportion (54–92%) of the variance remained unexplained. This may be due to the fact that these peptides are also produced by peripheral organs, and the source cannot be identified [33].

Our findings support the results showing lack of significant differences in plasma Aβ42 levels in cognitive norm subjects compared to subjects with sporadic AD [34,35,36,37], as well as the latest longitudinal studies reporting no differences in plasma concentrations of free Aβ42 between subjects with preclinical AD and normal cognition [38]. Recent meta-analyses also generally conclude that plasma concentrations of Aβ42 or Aβ40 in patients and controls remain the same. If altered, so that higher concentrations are reported for AD, there is a significant overlap between the groups [39].

Concerning AD risk prediction in individuals without cognitive impairment, several studies find that high plasma Aβ42 levels, or a high Aβ42/Aβ40 ratio, indicate future dementia, while others find the opposite [25,40,41]. Meta-analysis of studies concerning the predictive value of plasma Aβ42 levels in regard with conversion from MCI to dementia, encompassing 4 longitudinal studies conducted on 244 subjects [38,42,43] suggests that only elevated levels of Aβ40 show weak correlation with the conversion. No significant relationships between concentrations of Aβ42 or Aβ42/Aβ40 ratio, and the progression were identified [44]. There are, however, reports on baseline plasma Aβ42 concentrations and Aβ42/Aβ40 ratio indicating this association, with the respective levels being significantly lowered in patients who developed AD in comparison with cognitively stable MCI individuals [45]. Conversely, findings on elevated Aβ42 plasma levels in both familial AD [34] and the preclinical phase of the disease [25] demonstrate that they decrease gradually with dementia progression and amyloid plaque formation [25,46]. In a Swedish study analyzing both amyloid isoforms in a sample of 719 subjects, elevated plasma Aβ concentrations were linked to white matter lesions, brain micro-bleedings, hypertension, diabetes, and vascular diseases such as ischemic heart disease. However, they were significantly lower in demented patients [47]. This suggests that observations of changes in plasma Aβ42 dynamics could potentially enable the assessment of risk of dementia development. However, the latest findings from longitudinal studies on over 300 elderly participants contradict the above, reporting that plasma levels of free Aβ40 and Aβ42 did not alter significantly between preclinical AD (on average 9.3 years prior to clinical diagnosis) and cognitive norm. Respective levels did not help predict AD clinical progression, nor did they change over the years prior to dementia diagnosis in this sample [48]. Findings from the present preliminary study cannot be compared to the above; differences in study design, scope, cohort (our study cohort was limited to elderly Polish individuals), methods, and challenges in measuring Aβ levels, and, finally, their biological variation, all contribute to the discrepancies. The potential role of plasma Aβ levels as an early cognitive deterioration marker requires further empirical verification through prospective studies with more comprehensive scope. In addition, both the effects of patient variables on biomarker alterations, and the relationship between Aβ42 and clinical presentation, need to be investigated [8]. Our findings might, however, act as groundwork for further scientific exploration. Several cohort studies using large samples of well characterized participants, such as the Australian Imaging, Biomarker and Lifestyle (AIBL) Study and the Alzheimer’s Disease Neuroimaging Initiative (ADNI), have already made considerable advances in this area, including correlation with amyloid PET imaging and detailed clinical and laboratory follow-up. Most investigators have measured Aβ40 as well as Aβ42, and also looked at the Aβ42/Aβ40 ratio as a possible predictor. Large panels of plasma biomarkers have also been studied. Recently, neurofilament light (NfL) has been suggested as a candidate biomarker.

There are multiple challenges to measuring Aβ levels. They vary with the time of day and with fasting status. Moreover, Aβ has much lower levels in plasma than in CSF [42,47]. Interestingly, the recent discovery of hypersensitive methods, namely SIMOA and immunoprecipitation-mass spectrometry (IP-MS), might enable detection of slight Aβ plasma level alterations in subjects with early cognitive decline. Precise quantitation of plasma Aβ42 level to sub-picograms per millimeter was achieved with SIMOA [49]. It was proven to quantitate Aβ40 and Aβ42 levels in blood plasma and CSF of 274 cognitively normal, 174 subjective cognitive decline (SCD), 214 MCI, and 57 AD individuals [47]. Results showed lowered concentrations of Aβ42 in the plasma of AD patients as opposed to the control group. In subjects with pathological CSF levels, Aβ42 plasma concentrations lowered progressively in SCD, MCI, and AD, respectively compared to cognitive norm. AD patients with pathological CSF signature were, however, the only group presenting significant differences in plasma Aβ42 from controls. This result implies a constrained power of this biomarker for distinguishing early cognitive decline with CSF pathologies. Plasma composite biomarker showed a strong relationship between plasma and CSF concentrations amongst AD subjects, demonstrating an equivalent diagnostic power to CSF Aβ42 in determining Aβ concentrations [50]. Therefore, recent advances in measurement techniques, such as IP-MS, may hopefully result in better utility [51,52,53].

Currently, due to the inconsistency of the findings, it can be assumed that plasma Aβ does not reflect brain Aβ turnover or metabolism [54] well enough to allow the prediction of the disease progression [38]; therefore, it might not be a good candidate for an early neurodegeneration biomarker. With the appearing hypersensitive assays, plasma Aβ has the potential to become an appropriate biomarker in conjunction with other diagnostic methods. The discrepancy of results of Aβ as a blood biomarker of neurodegeneration, as well as the insignificant changes of Aβ42 concentrations in blood plasma, have also been recently connected to Aβ epitope masking by its binding to plasma proteins, which is a limitation of ELISA or other standard immunoassays. It is worth noting that this weak linkage with the disease pathology may be an effect of Aβ contribution from peripheral tissues to plasma, as also evidenced by the lack of association between respective plasma and CSF levels [42]. It can therefore be a derivative of limited analytical power of typically used immunoassays or ELISA [55], or other disturbances that could be reduced by test enhancements in the near future [56].

Limitations to this preliminary study should be considered; only routine screening examination of cognition, mood, and functional dependence was performed. No further assessments have been made, including brain imaging data. Additionally, our subjects were Polish elderly, therefore the results cannot be generalized to other populations. Despite limitations, it is an important contribution to the research investigating amyloid beta as a potential plasma biomarker of an early cognitive deterioration in older adults.

## 5. Conclusions

Based on our preliminary study, we can assume that amyloid beta needs to be studied further. Future courses of action, involving more candidate biomarkers and utilizing alternative detection methods on larger cohorts might potentially accelerate the development of innovative strategies to enhance the prognosis of brain disorders. Therefore, the role of plasma Aβ as a marker of an early cognitive deterioration requires further research. Although it was initially thought to be a promising candidate, a great deal of research over more than 20 years has not yet been as fruitful as was hoped. However, recent advances in the area may result in better utility and bring hope that plasma Aβ has the potential to become an appropriate biomarker in conjunction with other diagnostic methods.

## Figures and Tables

**Table 1:** Demographic Characteristics of the Population.

Cognitive Status	Cognitive Norm			MCI			Mild Dementia			Total		
Age	F	M	T	F	M	T	F	M	T	F	M	T
66–74	13	13	26	17	14	31	7	13	20	37	40	77
75–85	12	15	27	11	15	26	12	14	26	35	44	79
85+	8	10	18	15	13	28	14	14	28	37	37	74
Total	33	38	71	43	42	85	33	41	74	109	121	230

Sample characteristics (F—female, M—male, T—total).

## Data Availability

The authors confirm that the data supporting the findings of this study are available within the article.
